# Is *β*-Hydroxybutyrate a Magic Pill in CKD?

**DOI:** 10.34067/KID.0000000876

**Published:** 2025-07-31

**Authors:** Jing Liu, Frank C. Brosius, Jing O. Wu

**Affiliations:** 1 Department of Physiology, College of Medicine – Tucson, University of Arizona, Tucson, Arizona; 2 Division of Nephrology, Department of Medicine, College of Medicine – Tucson, University of Arizona, Tucson, Arizona

**Keywords:** Alport syndrome, CKD, GFR, metabolism, mortality, renal fibrosis, renal function decline, renin angiotensin system, SGLT2

Multiple large clinical trials have demonstrated that renin-angiotensin system (RAS) and sodium-glucose transporter 2 (SGLT2) inhibitors, especially in combination, slow the decline of GFR and markedly reduce morbidity and mortality in patients with CKD.^1^ Despite these advances, the loss of kidney function in patients with CKD treated with RAS and SGLT2 blockade still exceeds the age-related GFR decline in non-CKD individuals.^2^ Residual inflammation and fibrosis may drive continued nephron loss, even with these recommended treatments, suggesting that additional therapeutic interventions are needed to further improve outcomes.

**Figure 1:**
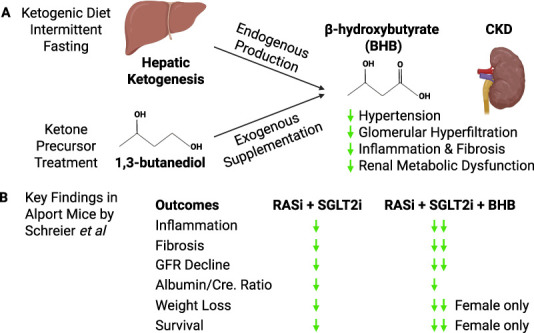
**Renoprotective effects of BHB.** (A) BHB is an endogenous product of hepatic ketogenesis, which is increased by ketogenic diet and intermittent fasting. Exogenous BHB can be supplemented through intake of its precursor 1,3-butanediol. BHB exhibits renal protective effects in CKD and proposed mechanisms include reduction of hypertension, alleviation of glomerular hyperfiltration, anti-inflammatory and antifibrotic effects, and correction of renal metabolic dysfunction. (B) Observed renal protective effects of BHB precursor treatment in combination with dual RAS/SGLT2 inhibition therapy in a mouse model of Alport syndrome by Schreier *et al.* Compared with dual RAS/SGLT2 blockade, BHB supplementation decreased inflammation and fibrosis and slowed GFR decline but failed to further decrease albumin-to-creatinine ratio. For reasons yet to be understood, the renoprotective effects of BHB supplementation selectively reduced weight loss and mortality in female, but not male, Alport mice. BHB, *β*-hydroxybutyrate; Cre., creatinine; RAS, renin-angiotensin system; RASi, RAS inhibition therapy; SGLT2, sodium-glucose transporter 2; SGLT2i, SGLT2 inhibition therapy.

For some time, ketogenic diets and ketone precursor treatment have been identified as potentially therapeutic in multiple diseases including CKD (Figure 1). The study by Schreier *et al.* found that addition of oral 1,3-butanediol, a precursor of *β*-hydroxybutyrate (BHB), to a regimen of ramipril and empagliflozin therapy enhanced kidney protection.^3^ Treatment with 1,3-butanediol in addition to the other two agents led to slower GFR decline, reduced BUN, and attenuated renal fibrosis more than RAS and SGLT2 inhibitor therapy alone in a genetic mouse model of Alport syndrome. These results add to the literature that ketogenic therapy could be a promising approach to slow progression of CKD and improve outcomes even in those on current state-of-the-art treatment.

Under physiological conditions, renal autoregulation protects nephrons from barotrauma and ischemic injury by maintaining constant renal blood flow and glomerular filtration over a wide range of BP. In early hypertension and diabetes, impaired renal autoregulation exposes the nephrons to systemic hemodynamic pressure, leading to glomerular injury, proteinuria, and nephron loss.^4^ Glomerular hypertension can continue to damage remaining nephrons, causing progressive glomerulosclerosis, GFR decline, and ultimately ESKD. In this process, glomerular hypertension and hyperfiltration can also induce chronic inflammation characterized by macrophage infiltration and upregulation of chemokines and profibrotic inflammatory cytokines. Many of these processes also play an important role in CKD progression independent of the hemodynamic changes. For example, increased fatty acid oxidation and glucose metabolism occur in proximal tubules in diabetes and stimulate intracellular signaling through enhanced activity of the mammalian target of rapamycin complex 1 that drive both inflammatory and fibrotic responses in humans with diabetic nephropathy.^5^

In CKD, kidney protective effects of combined RAS and SGLT2 blockade have been attributed to a number of effects including improved BP control, reduction of oxidative stress, suppression of inflammation and fibrosis, and improvement of cardiovascular function.^6^ Both of these therapies attenuate glomerular hyperfiltration, although through different mechanisms. SGLT2 blockade ameliorates glomerular hyperfiltration by increasing afferent arteriolar resistance, a combined effect likely achieved by correcting hyperglycemia-induced preglomerular vasodilation and blunted tubuloglomerular feedback in diabetes,^7^ whereas RAS blockade is thought to mitigate glomerular hyperfiltration through a net reduction of efferent arteriolar resistance and consequent decrease in glomerular capillary pressure.^8^ Because BHB also reduces BP,^9^ it may also have direct effects on afferent and efferent arterioles. SGLT2 inhibitors also reverse multiple pathogenic effects independent of the hemodynamic effects including the altered proximal tubule metabolism, reduction in mammalian target of rapamycin complex 1 activity, and tubular injury.^5^ RAS inhibitors have direct tubular effects, and both SGLT2 and RAS inhibitors have been shown to have anti-inflammatory and antifibrotic effects. Similarly, ketosis or ketone supplementation has been found to have multiple metabolic anti-inflammatory and antifibrotic effects.

BHB is generated by the liver, and its production is decreased in response to high salt intake.^9^ Reduced BHB results in increased renal NLR family pyrin domain containing 3 inflammasome activity and salt-sensitive hypertension, while BHB supplementation lowers hypertension and attenuates hypertensive kidney injury. Interestingly, dapagliflozin promotes BHB production in streptozotocin-induced diabetes, suggesting that the renoprotective effects of SGLT2 inhibition could be mediated in part by the anti-inflammatory and antifibrotic effects of BHB.^10^

Previous studies have examined the effect of 1,3-butanediol on CKD models in rodents. Administration of 1,3-butanediol preserved kidney function and downregulated inflammatory responses in male Dahl salt-sensitive rats on a high-sodium diet,^9^ and a subsequent study of female rats in the salt-sensitive spontaneous hypertensive rat strain, another hypertensive rat model that develops kidney disease, found that 1,3-butanediol administration was similarly protective and reduced markers of proximal tubular injury.^11^

In the study by Schreier *et al.*, 1,3-butanediol supplementation on top of dual RAS/SGLT2 blockade generated enhanced antifibrotic effects. This was demonstrated by decreases in profibrotic cytokine production, TGF*β*1-SMAD3 signaling, and fibronectin expression. However, effects of 1,3-butanediol supplementation in their study did not translate into statistically significant survival benefits when male and female data were combined. Oddly, BHB in combination with dual RAS/SGLT2 blockade achieved statistically significant survival benefit only in female, but not male, Alport mice (Figure 1). Previous studies have not found such sex differences, and additional studies are warranted to investigate the molecular basis of this sex difference in BHB responsiveness.

While the study by Schreier *et al.* adds meaningfully to our understanding of the potential role of ketosis or ketone precursor supplementation in adding to the multiple kidney protective effects of combined SGLT2 and RAS inhibition, the mechanism remains unclear. Given the public embrace of intermittent fasting and other methods to induce ketosis or take supplements that increase BHB, it will be important to fully understand the protective effects as well as potential downsides of such treatments before advocating such therapy for kidney disease treatment and prevention.
